# Remote Monitoring Empowerment of Patients with IBDs during the SARS-CoV-2 Pandemic

**DOI:** 10.3390/healthcare8040377

**Published:** 2020-10-01

**Authors:** Mauro Mastronardi, Margherita Curlo, Maurizio Polignano, Natalino Vena, Daniela Rossi, Gianluigi Giannelli

**Affiliations:** National Institute of Gastroenterology, “Saverio de Bellis” Research Hospital, 70013 Castellana Grotte, Italy; mauro21mastronardi@gmail.com (M.M.); margherita.curlo@irccsdebellis.it (M.C.); maurizio.polignano@irccsdebellis.it (M.P.); natalino.vena@irccsdebellis.it (N.V.); daniela.rossi@irccsdebellis.it (D.R.)

**Keywords:** smart phone, tele-monitoring, inflammatory bowel disease (IBD), Crohn’s Disease (CD), Ulcerative Colitis (UC)

## Abstract

Once the WHO declared the Sars-CoV-2 pandemic, the world had to reprogram numerous clinical activities, particularly those related to highly disabling diseases such as inflammatory bowel diseases (IBDs). In this study, 1083 IBD patients were assessed, affected by Crohn’s Disease (CD) and Ulcerative Colitis (UC), and subdivided into two groups. The first group included patients who needed treatment in person at the outpatients clinic, while the second group could be tele-monitored because they were able to self-administer therapy. The tele-monitoring was based on telecommunication applications via smartphone, driven by a dedicated clinical control room in the IBD Clinic. The aim of this study was to assess the quality of life (using IBDQ32) of UC patients and tele-monitored CD patients (tele-monitoring group) as compared to those patients who underwent assessment in person in the outpatients clinic (control group). Despite observing a lower number of relapses in the control group than the tele-monitoring group, there were no statistically significant differences between the groups in terms of the IBD32Q scores. Tele-monitoring of patients who are able to self-administer the IBD therapy can be an effective vicarious system as compared to the clinical evaluation in person, that could lead to important changes to avoid the overcrowding of the IBD outpatients clinic, especially during public health crises like the present pandemic.

## 1. Introduction

The outbreak of SARS-CoV-2 was first reported in Wuhan, China, but rapidly spread worldwide, and has caused approximately 13,150,645 cases and 574,464 deaths (updated to the 15th of July 2020), as declared by the WHO [1]. The virus is extremely infectious, and Italy, unfortunately, as the first European country to be affected, has also experienced one of the most severe epidemics, with 227,421 cumulative cases in which 28.6% of patients had to be admitted to the intensive care unit and 14.4% of the total cases died [2]. The critical situation obliged the Italian Government to adopt several measures to contain the spread of the infection, including social distancing, lockdown of all non-vital activities, and community isolation. Routine medical activities, such as inpatients activities for patients with inflammatory bowel diseases (IBDs), were reduced, both for patients’ safety reasons and to avoid a possible explosion of the contagion. IBDs are disorders of multifactorial causes that present in a multitude of phenotypes, with different clinical behaviors and severity. IBDs cover a spectrum of disorders that consist of a focal inflammation of the gut with different clinical manifestations. Crohn’s Disease (CD) and Ulcerative Colitis (UC) are considered as the two extremes of this spectrum [3]. UC is characterized by long-lasting inflammation and sores (ulcers) in the innermost lining of the large intestine (colon) and rectum [3]. Crohn’s Disease is characterized by more diffuse inflammation of the lining of the digestive tract, which often spreads deep into affected tissues [3].

Common clinical manifestations of both UC and CD are severe diarrhea, abdominal pain, fatigue, and weight loss. Those types of IBDs can be debilitating and sometimes lead to life-threatening complications. The prevalence of these diseases in the south of Italy is about 126–158 per 100,000 inhabitants for UC and 142–178 per 100,000 inhabitants for CD [4]. Patients with IBD require constant monitoring of their disease and rapid adjustment of the pharmacological treatment regimen in cases of relapse, to improve the clinical outcome and prevent further complications. In addition, anxiety and other behavioral disorders are associated with IBD, and it is well known that this leads patients to overestimate their symptoms [5]. It is also well known that contact with medical staff involved in the treatment and monitoring of IBDs could be crucial to better weigh up the real contribution of emotivity to the disease conditions; thus, possibly reducing the number of disease relapses [6]. The aim of this study was to assess the quality of life (QoL) of UC and CD tele-monitored patients compared to those patients who underwent assessment in person in the outpatients clinic.

## 2. Materials and Methods

### 2.1. Study Population and Design

Herein we report a single-center cross-sectional study conducted in the clinical setting of the IBD tertiary referral center, at the National Institute of Gastroenterology, Research Hospital “Saverio de Bellis”, located in Castellana Grotte (Bari), Italy. Between 9 March and 24 April 2020, we selected 1038 IBDs patients from those already followed by the clinicians, who should have been visited by the medical staff in that period, as scheduled before the Sars-CoV-2 lockdown imposed as of 8 March 2020 in Italy. We subdivided the sample into two groups: (1) 421 patients assessed in person, undergoing different therapy protocols (they needed infusions with biological drugs for CD or UC), defined as the control group; (2) 617 patients who did not need treatment in person (i.e., able to self-administer therapy), defined as the tele-monitoring group. Patients who underwent both types of assessment were excluded from the data analysis. This observational study was approved by the IRCCS “Saverio De Bellis” Institutional Review Board (n.333/18-20).

### 2.2. Remote Monitoring Devices

Our group offered support by phone, videoconference, made with the technological support of our ITC team and smartphone teleconference software. This tele-monitoring was scheduled every three days, by remote video call and/or other remote support (i.e., telephone call). The calls were managed by clinical IBDs staff from the dedicated control room. Both categories of IBDs patients (CD and UC) received remote support using different channels, depending on their ability and the type of technological support at their disposal at home. The entire sample was assigned to one of the two groups of patients: (1) all the subjects who needed biological drug treatment administered by infusion, and for this reason had to be assessed in the outpatient clinic; (2) patients who could take oral or subcutaneous self-administered therapy (e.g., corticosteroids, 5ASA, oral immunosuppressors, etc.).

### 2.3. Clinical and QoL Assessments

At the baseline, from January to March 2020, before the national lockdown was declared (8 March 2020) we recorded only general characteristics of the subjects. After 2 months (from the 1st to the 15th of May) both groups of patients were remotely assessed with the Mayo Score for UC assessments [7] and the Harvey–Bradshaw Index (HBI) score [8] for CD. To assess the quality of life (QoL), we used a well validated questionnaire, the IBDQ32, based on “subjective nonclinical” items, that could be administered by telephone. The IBDQ32 can make full measurements of the QoL and offer partial scores that discretely analyze bowel, systemic, emotional and social domains related to the IBDs [9]. We registered any occurrence of one or more relapses of the disease (Relapse ≥ 1) during the 60 days of observation period. The average number of remote or in person follow-up visits was calculated for each patient during the 60 days of observation during lockdown.

### 2.4. Statistical Analysis

We performed statistical analysis of baseline variables as the mean ± standard deviation (SD), median, and range for continuous variables and proportion (%) for the frequency of categorical variables. The sample (N = 1038) was subdivided into 2 groups: the control group (421 subjects treated at the hospital) and the tele-monitoring group (617 subjects monitored at home). Since we had no previous study data with the same aim, we considered this study as a pilot study, and could not calculate a sample size.

The normality of distribution was assessed for each variable using Shapiro‘s test. All variables resulted normally distributed. For this reason, parametric analysis (independent *t*-test) was performed to assess mean differences of continuous variables between the two groups. The chi-squared test was used to assess differences in terms of proportion between categorical variables p-values less than or equal to 0.05 were considered statistically significant, with 95% confidence intervals. As a result, that we had no baseline assessment, due to the unpredictable onset of the pandemic, as outcome measure, we only performed a cross-sectional comparison between the groups for the QoL measurements. In addition, linear regression analysis was performed to assess the association, using QoL as dependent variable, the control group as intercept (0), and the intervention as covariate (1). To assess the known confounding effect, in the models we implemented the covariates: CD\UC, number of relapses, average number of follow-up visits to test the confounding effect on the QoL. Additional analysis was made of the sub-totals of the IBDQ32 scores. We only performed a cross-sectional comparison between the groups for the QoL measurements. All statistical analyses were performed using RStudio Team (2020). RStudio: Integrated Development for R. RStudio, PBC, Boston, MA, USA.

## 3. Results

### General Characteristics and Groups Comparison

In the IBD Unit at our center, in total, 1038 patients were selected for follow-up. None of the patients had symptoms related to the SARS-CoV-2 infection. The mean age of the whole sample was 41.23 years, SD 2.4, and 45% were male. Among the entire sample, 421 (41%) received intravenous biological drugs (e.g., infliximab, vedolizumab, and a first infusion of Ustekinumab) so they needed to come to the Center for the intravenous drug infusion. The remaining 617 (59%) patients needing pharmacological therapy were defined as the tele-monitoring group, because they underwent close clinical tele-monitoring. The general characteristics of the two groups are shown in Table 1. There were significantly more relapses in the telemonitoring group compared to the control group. In fact, during the observation time, among the tele-monitoring group subjects, 23.6% developed at least 1 episode of recurrence of their disease, assessed in remote mode. In the control group, 18.3% suffered more than 1 episode of recurrence. Among the subjects who underwent relapse, in the tele-monitoring group, 61.3% were affected by UC and 38.7% by CD (*p* < 0.05). Among the patients with relapse in the control group, 58.8% were affected by UC and 41.2% by CD. Moreover, the subjects in the telemonitoring group had significantly higher Mayo and Harvey–Bradshaw scores (*p* < 0.05). Despite that, no significant differences in the total IBD32Q score were observed between the tele-monitoring group and the control group (*p* > 0.05), as shown in Table 1. Table 2 and Table 3 show the differences in the different domains of the QoL between the CD and UC groups, subdivided into the tele-monitoring and control groups. The tables clearly show that the UC patients had higher general and partial IBD32Q scores than the CD patients, except for the Social domains. In addition, in Table 4, we show a linear regressive model, implemented to explore whether belonging to the tele-monitoring group rather than to the control group could be associated to increased scores at the IBD32Q questionnaire. In this model, adjusted for age, sex, CD, UC, Harvey–Bradshaw Index, Mayo Score, number of relapses, we did not find any significant association. Table 5 shows the sub-total scores for the IBD32Q: surprisingly, Emotional and Social scores were significantly higher in the tele-monitoring group than the control group.

## 4. Discussion

We report a first experience of the management of patients with IBDs using tele-monitoring technology, which was essential to ensure proper control of the disease, as suggested by the STRIDE10 study [10], during the Italian lockdown although we were not able to use the inflammatory markers suggested in the CALM study [11] because of the abrupt suspension of clinical visits at the start of the lockdown. The major finding of this study suggests that tele-monitoring does not differ, in terms of QoL, from the examination in person. In fact, among the two groups, we did not observe significant differences in subjective QoL after the Italian lockdown.

This finding seems to be fundamental during this crisis time, because patients could not undergo the classic therapeutic protocols, with follow-ups in person, so a strategy to substitute this type of clinical path was urgently needed. Particularly, the use of tele-monitoring systems, even with poor technology or consumer devices (i.e., video-call and chat) should be encouraged to deal with IBDs management in this particular period. Moreover, the rapid treatment of clinical recurrence is an essential element in the management of these patients, especially during this pandemic, when problems of anxiety, already inherent in this group of patients, were particularly severe [12]. This could determine the overlap of functional symptoms; such conditions, alongside the absence of objective evaluation data on the entity of the clinical relapse, made the remote clinical management of relapses extremely difficult. In our study, the QoL of tele-monitored patients did not show significant differences as compared with the traditional management in person of patients, that has resumed since the re-opening after lockdown. Nevertheless, UC patients seem to have a better overall QoL, in both the tele-monitoring and control groups, as compared to CD patients, particularly in the emotional dimensions of the questionnaire administered. This is probably due to the important components of behavioral determinants (i.e., anxiety, depression, loneliness) that usually coexists in CD patients [6].

Surprisingly, there were more relapses in the telemonitoring group, with higher MAYO and HBI scores, although this is probably due to differences in terms of severity of the disease. In fact, tele-monitored patients were usually affected by more severe symptoms compared to the control group, that mostly had mild to moderate symptoms. Moreover, the lack of physical control in the telemonitoring group could have led an underestimation of clinical symptoms and consequently to more relapses of the clinical manifestations. This situation could have been aggravated by the impossibility to sample and analyze fecal (calprotectin) and blood samples (C-Reactive Protein). This impossibility was determined by the Italian Government-imposed limitations of non-urgent clinical assays during the Covid-19 national crisis. Despite that, the quality of life, measured by the IBDQ32, was higher in the tele-monitoring group. When we analyzed the single sub-scores in the questionnaire (Table 5), we can see that on average the tele-monitoring group had lower scores for physical symptoms, compensated by better social and emotional scores. The reason for this may lie in the influence that a hospital setting can have on a psychologically sensitive patient with IBD. The improvement in QoL could be attributed to the absence of hospital contacts, reducing stress for the patients in the tele-monitoring group. On one hand, the lack of clinical visits in person could only partially be replaced by the remote video assistance needed to avoid the potential risk of Covid-19 infection, and it is clear that direct interaction between clinicians and patients heavily affects the mental health and the depressive status of these patients, with downstream effects on the individual lifestyle and quality of life that are so important for the subjective patient outcome [13]. On the other hand, the possibility to contact clinicians “on demand” through the video assistant likely contributed to ensure the maintenance of the QoL parameter even in the absence of direct, classical interaction with the physician, in the overwhelmingly difficult situation of the SARS-CoV-2 pandemic.

### Limitations and Strength

This study has several limitations, partly due to the short timeframe of the Covid-19 crisis: (1) the high heterogeneity of the groups (different treatments, diseases, and stages), that firstly introduced substantial bias of the results and secondly made the comparison difficult to generalize to another clinical setting; (2) in particular, the cross-sectional nature of the study, that cannot generate any causal inference; (3) the absence of baseline analysis of QoL, Mayo, and Harvey–Bradshaw Scores, that makes it impossible to apply appropriate longitudinal analysis; (4) the lack of availability of a correct description of phenotypic data at the time of the analysis of this study, because the clinical records were scattered due to their use in remote mode; (5) the absence of QoL and clinical data (i.e., relapse numbers) before the outbreak for each subject.

The most important strength of this study is that it was planned and administered over a short period during the unprecedented crisis event of the Covid-19 pandemic.

## 5. Conclusions

Further studies are needed to evaluate how best to engage patients with telemedicine platforms and to identify those subjects who may be well managed with such technological approaches. Particularly, a well-designed clinical trial should be used to compare tele-health management in contrast to traditional care of IBD patients in person, using robust longitudinal non-inferiority analysis. This type of study should be supported by integrated, specifically designed tele-health systems, dedicated to the care of IBD patients. One of the future practical measures could be the implementation of a more complex home-based IBD patients setting. In particular, the use of portable calprotectin assay systems for fecal samples and rapid tests for inflammatory markers on peripheral blood may be useful in a completely home-based examination protocol [14].

## Figures and Tables

**Table 1:** Summary of patients’ characteristics by group: control and tele-monitoring groups.

Variables	Controls		Tele-Monitoring		(*p*-Value)
	(Mean ± SD)	Median/Ranges	(Mean ± SD)	Median/Ranges	
Age	43.6 (±3.1)	42.8/35–56	44.1 (±4.5)	44/38–55	0.51
Men	172 (41%)	-	271 (44%)	-	0.06
CD (%)	164 (38.9)	-	254 (41.6)	-	0.05
UC (%)	257 (61)	-	361 (58.5)	-	0.06
% Relapses ≥ 1	77 (18.3)	-	145 (23.5)	-	0.02
Mayo (for UC patients)	4.3 (±3.7)	4.2/0–8	5.3 (±4.4)	5.2/0–8	0.02
Harvey–Bradshaw Index (for CD patients)	10.5 (±4.1)	10/2–30	12.5 (±3.9)	10/2–25	0.02
IBDQ32	167.6 (±39.6)	162/40–182	158.4 (±35.3)	156/38–180	0.89
Average Numbers of Follow-up *	3.6	3/2–4	3.8	3/2–4	0.09

* Average numbers of follow-ups for each group over the two months period of observation.

**Table 2:** Total and partial IBDQ32 scores for the tele-monitoring group.

Sub-Scores	CD	UC	(*p*-Value)
	(Mean ± SD)	(Mean ± SD)	
Bowel	38.6 (11.2)	59.1 (10.4)	0.03
Systemic	19.3 (7.4)	27.9 (6.5)	0.04
Emotional	42.2 (15.9)	61.9 (15.4)	0.03
Social	28.5 (8.1)	29.4 (7.1)	0.46
Total	146.6 (39.6)	178.4 (35.3)	0.03

**Table 3:** Total and partial IBDQ32 scores for the control group.

Sub-Scores	CD	UC	(*p*-Value)
	(Mean ± SD)	(Mean ± SD)	
Bowel	36.1 (12.1)	58.6 (11.3)	0.04
Systemic	20.4 (6.1)	28.7 (7.9)	0.04
Emotional	40.1 (16.8)	60.3 (18.1)	0.03
Social	30.1 (9.3)	29.5 (9.7)	0.48
Total	157.6 (39.6)	178.4 (35.3)	0.03

**Table 4:** Linear regression analysis of IBDQ32 as dependent variable, the tele-monitoring group (as principal regressor) compared to the control group.

	β *	C.I. (95%)	(*p*-Value)
Tele-Monitoring Group	−3.6	−4.11 to 0.93	0.06
CD\UC	1.6	−0.22 to 3.61	0.61
Harvey–Bradshaw Index	2.9	−0.01 to 10.12	0.92
Mayo Score	−0.19	−0.69 to 0.11	0.16
Relapses	−0.1	−0.01 to 1.12	0.06
Follow-up	−1.7	−0.25 to 0.21	0.01

* β is the regression coefficient estimate adjusted for age, sex, Crohn’s Disease (CD)/Ulcerative Colitis (UC), Harvey–Bradshaw Index, Mayo Score, Number of Relapses.

**Table 5:** Total and partial IBDQ32 scores for the tele-monitoring vs. control group.

Sub-Scores	Tele-Monitoring	Control	(*p*-Value)
	(Mean ± SD)	(Mean ± SD)	
Bowel	41.7 (11.2)	56.8 (13.4)	0.06
Systemic	19.6 (16.3)	22.9 (9.9)	0.05
Emotional	60.4 (19.1)	48.6 (16)	0.03
Social	41.1 (2.1)	30.6 (9.5)	0.04
